# Supplementary material for: Targeting Insulin-Like Growth Factor 1 Receptor Delays M-Phase Progression and Synergizes with Aurora B Inhibition to Suppress Cell Proliferation
Source: Int J Mol Sci. 2020 Feb 5;21(3):1058. doi: 10.3390/ijms21031058 (PMC7037296; doi:10.3390/ijms21031058)
Supplement: Supplementary file 1 [file ijms-21-01058-s001.pdf]

Supplemental Figures

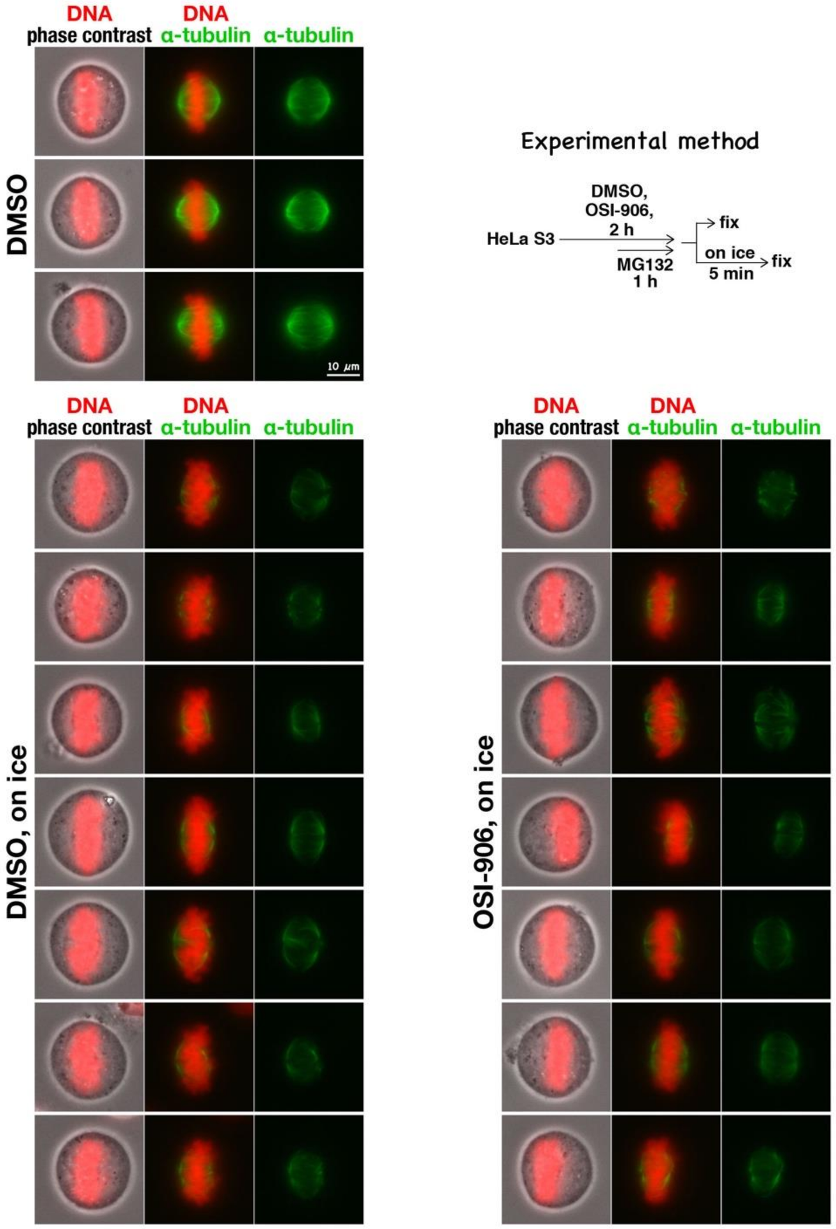

Supplemental Figure S1. OSI-906 does not reduce microtubule stability upon cold treatment.

HeLa S3 cells were treated with 3 μM OSI-906 or DMSO (solvent control) for 2 h. During the last 1 h, cells were simultaneously treated with 40 μM MG-132. The culture medium was changed to prechilled (in ice water) medium and the culture dishes were left on ice for 5 min. The cells were fixed in pre-warmed (37°C) PTEMF [4% formaldehyde, 2 mM PIPES (pH 6.8), 0.2% Triton X-100, 10 mM EGTA, and 1 mM $MgCl_2$] for 20 min at room temperature and stained for DNA (red) and α-tubulin (green). Cold treatment reduced the fluorescence intensity of α-tubulin, indicating the depolymerization of microtubules; however, no difference was observed in OSI-906-treated cells compared to solvent control.

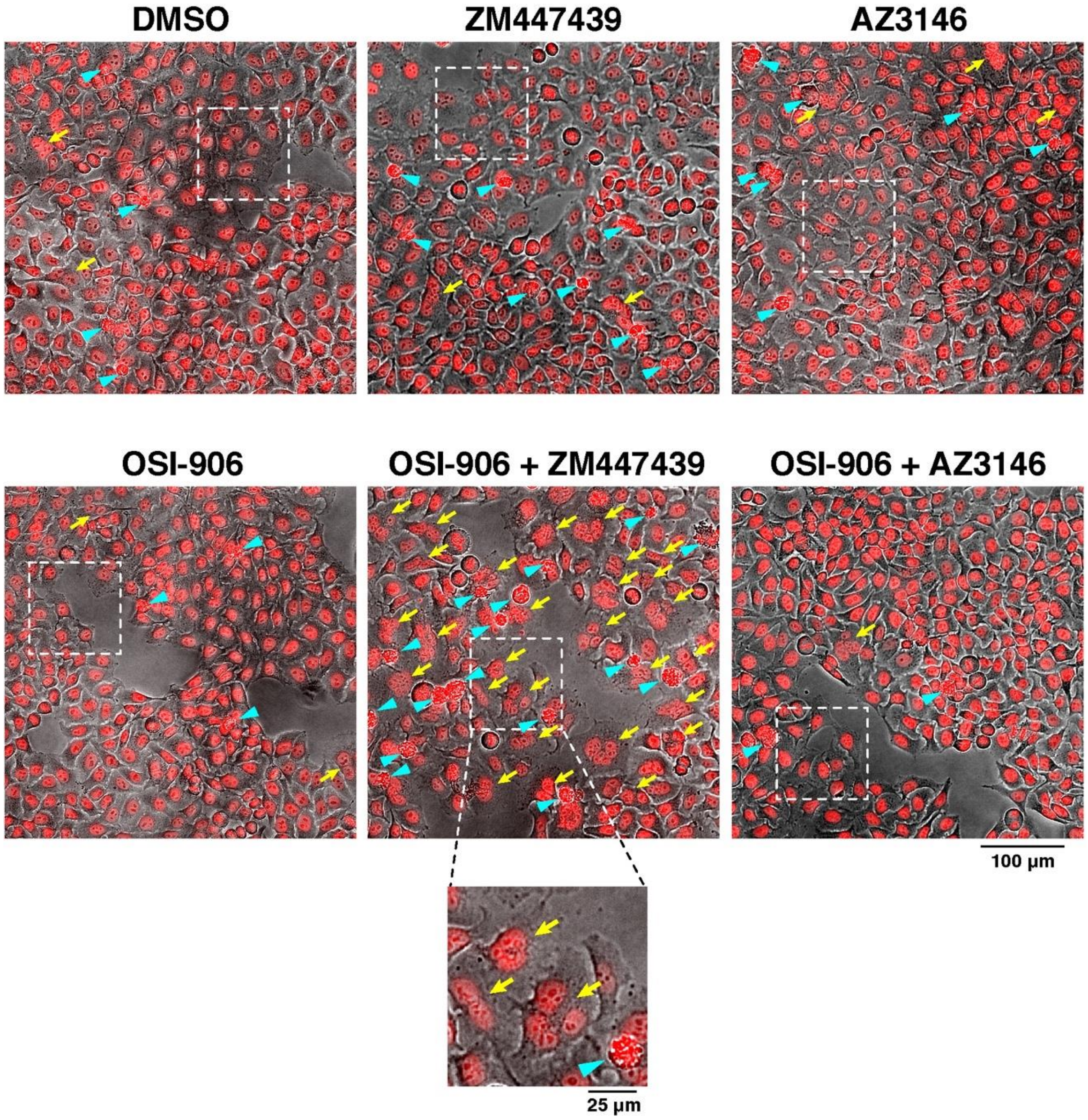

Supplemental Figure S2. Combination treatment of OSI-906 and ZM447439 causes multinucleation. Cells were seeded in a 24-well plate. After 51 h of treatment with inhibitors or DMSO, live cell nuclei were stained with 10 µM Hoechst 33342 (pseudo-colored red). After washing with PBS(–), cell images were captured. Yellow arrows indicate multinucleated cells. Blue arrowhead indicates dead cells. Boxed areas are shown in Figure 7E.

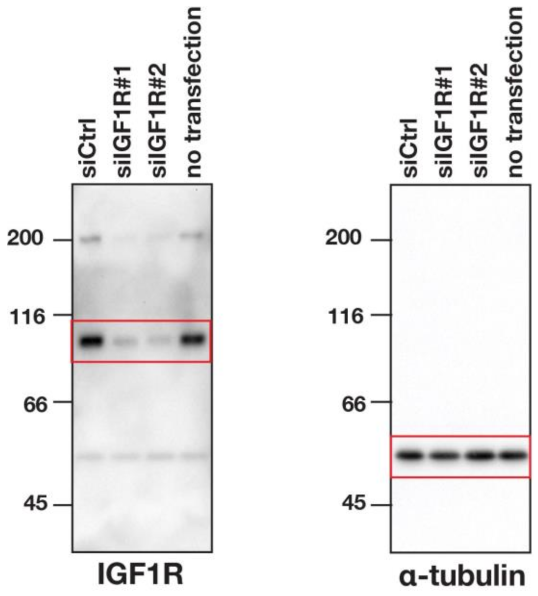

Supplemental Figure S3. Full-length blots for Figure 1.

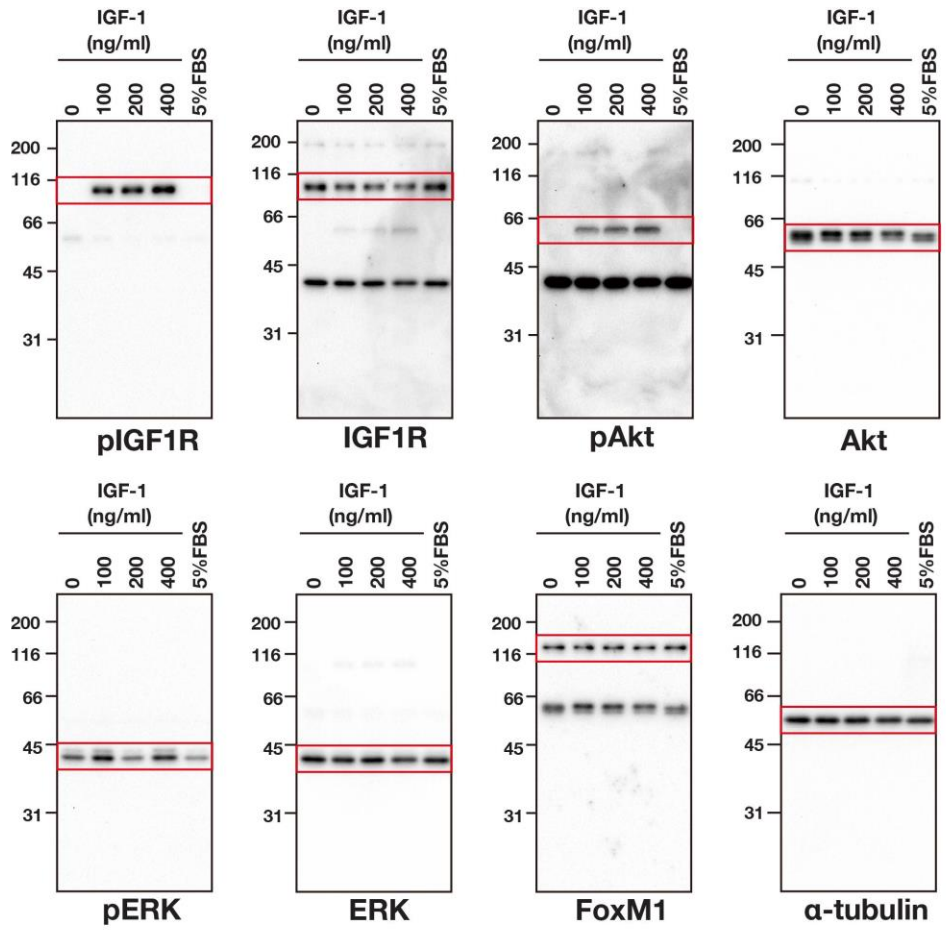

Supplemental Figure S4. Full-length blots for Figure 3.

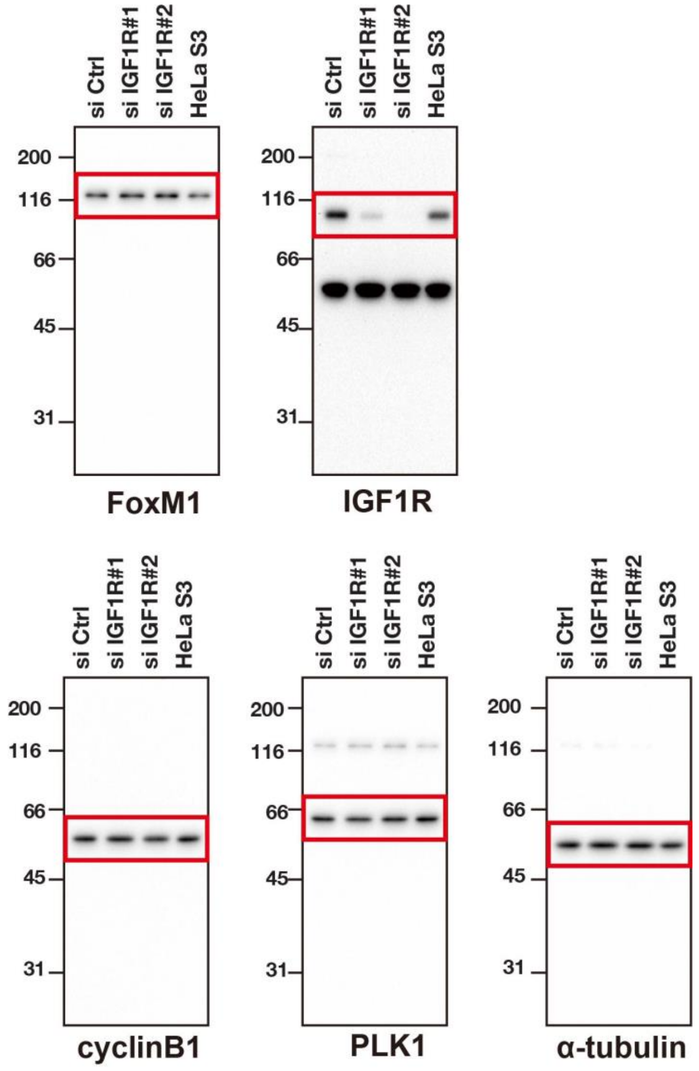

Supplemental Figure S5. Full-length blots for Figure 4.

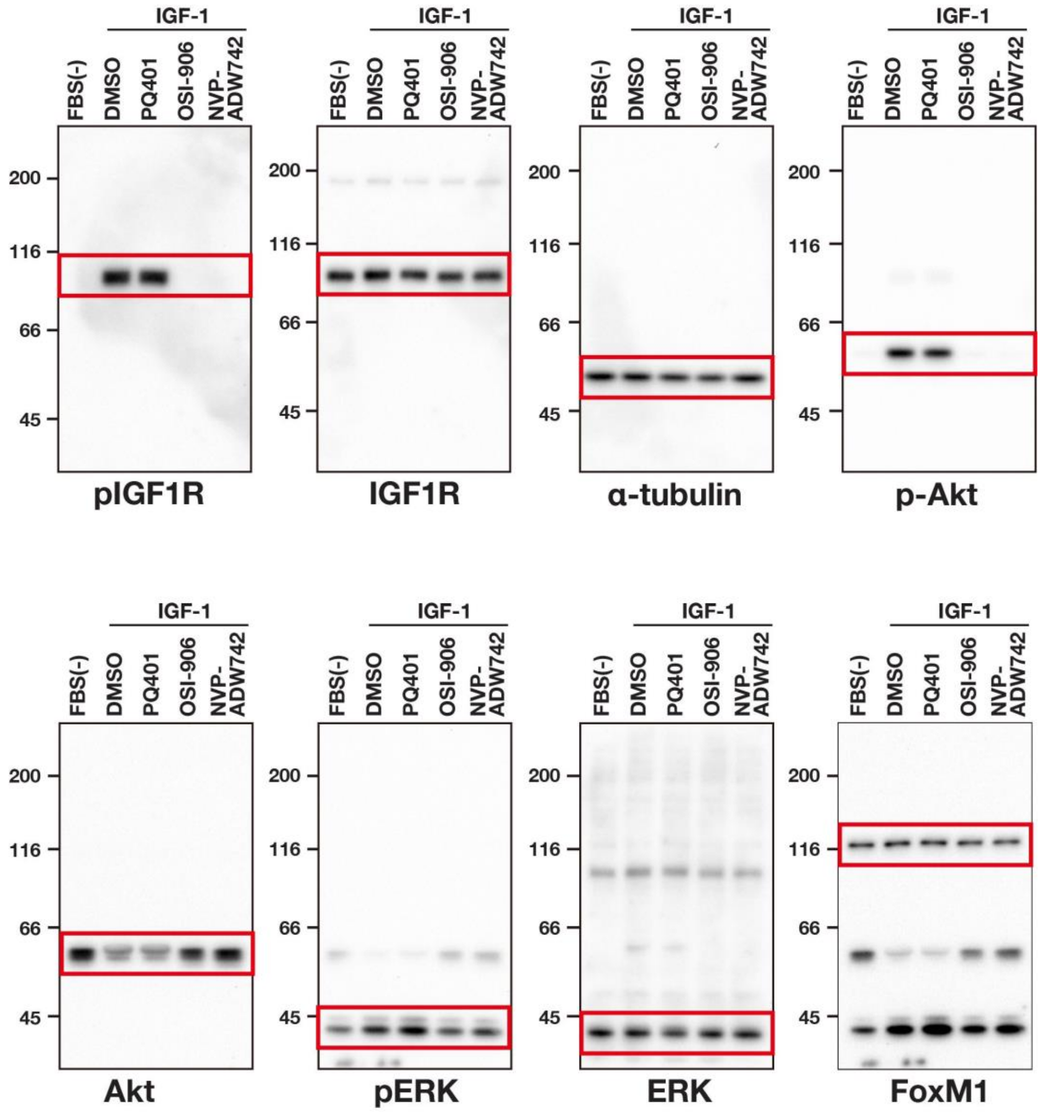

Supplemental Figure S6. Full-length blots for Figure 5.
